# Viscoelastic Effects on Drop Deformation Using a Machine Learning-Enhanced, Finite Element method

**DOI:** 10.3390/polym12081652

**Published:** 2020-07-25

**Authors:** Juan Luis Prieto

**Affiliations:** Escuela Técnica Superior de Ingenieros Industriales, Departamento de Ingeniería Energética, Universidad Politécnica de Madrid, José Gutiérrez Abascal 2, 28006 Madrid, Spain; juanluis.prieto@upm.es; Tel.: +34-91-067-7167

**Keywords:** drop, finite element method, machine learning, multiphase flow, particle level set, non-Newtonian fluid

## Abstract

This paper presents a numerical study of the viscoelastic effects on drop deformation under two configurations of interest: steady shear flow and complex flow under gravitational effects. We use a finite element method along with Brownian dynamics simulation techniques that avoid the use of closed-form, constitutive equations for the “micro-”scale, studying the viscoelastic effects on drop deformation using an interface capturing technique. The method can be enhanced with a variance-reduced approach to the stochastic modeling, along with machine learning techniques to reconstruct the shape of the polymer stress tensor in complex problems where deformations can be dramatic. The results highlight the effects of viscoelasticity on shape, the polymer stress tensor, and flow streamlines under the analyzed configurations.

## 1. Introduction

Bubble and drop dynamics in non-Newtonian fluids are a topic of undeniable interest within the community [1], owing largely to the number of real-world situations that may benefit from a comprehensive knowledge of the underlying physics: from drop formation mechanisms [2], to biomedical equipment involving droplet manipulation [3] or engineering devices in which breakup plays a central role [4,5], from droplet impact on liquid surfaces [6] to the study of drop dynamics within polymer gels and solutions [7,8]; a deeper understanding of this type of multiphase flows not only would improve existing manufacturing processes, but also encourage the development of new applications and spur breakthroughs in scientific research.

To study the multiphase flow of polymeric liquids, one should choose an appropriate discretization method capable of providing an accurate description of the interface. Mesh-based methods [9] offer such representation of the interface, either in an explicit (interface-tracking) or in an implicit (interface-capturing) form. Among the former, front-tracking and Arbitrary Lagrangian-Eulerian (ALE) schemes [10,11] display excellent performance in terms of mass conservation and shape preservation; however, remapping techniques are often found necessary [12] under extreme deformation of the moving interface. In contrast, interface-capturing methods follow a Eulerian approach, with the ‘Volume-Of-Fluid’ (VOF) method positioning itself as one of the most popular techniques within this context [13,14], showing good conservation properties, but requiring additional tools for handling geometrical quantities derived from the interface [15]. As an alternative, Level Set (LS) methods [16,17] capture the interface as the zero isocontour of a certain scalar function, which is advected by the flow, with noticeable mass loss and shape degradation if excessive diffusion is introduced during the advection stage. Despite these shortcomings, the LS method is widely used for interface problems undergoing dramatic deformation and topological changes and can be readily enhanced via “hybrid” schemes such as the Particle Level Set (PLS) method [18] that are able to ease, to a large extent, many of its drawbacks. Whatever the multiphase technique, the correct implementation of appropriate Boundary Conditions (BC) for the configuration of interest is a topic that requires careful consideration of the chosen discretization method [19], as it may have an enormous influence on the actual shape of the interface.

A common approach to handling the polymer interaction with the flow at a macroscopic scale is to represent the polymer contribution to the stress tensor by means of a closed-form, “constitutive” equation; thus, e.g., the work of Yue et al. [20,21,22] on drop deformation and complex two-phase flow using a diffuse-interface method and constitutive modeling; in [23], Pillapakkam employed an LS method to study rising bubbles in viscoelastic media, while Foteinopoulou and Laso [24] used a Phan–Thien-–Tanner model together with an elliptic mesh-deformation algorithm to investigate bubble oscillation; Castillo et al. proposed an LS method with a pressure-enriched FE space to study the two-fluid flow problem along with a Giesekus model for the polymeric liquid [25]; Fraggedakis et al. [26] characterized the critical volume of a bubble rising in a viscoelastic fluid using an FEM-based method and the exponential Phan–Thien and Tanner model; using a coupled LS-VOF (“VOSET”) method, Wang et al. [27] studied drag reduction in cavity flow; Xie et al. [28] focused on droplet oscillation under a Maxwell model using lattice Boltzmann techniques. In contrast to constitutive modeling, the “micro-macro” approach [29] tackles the polymer-flow interaction using stochastic and Brownian Dynamics (BD) simulations [30,31,32,33] to retrieve the polymer stress tensor from the internal configurations of the polymer particles advected by the flow. Taking the CONNFFESSITapproach of Laso and Öttinger [34], Cormenzana and co-workers [35] and later Grande et al. [36] successfully handled free surface flows of polymer solutions, while Prieto [7,37] conducted multiphase simulations in viscoelastic fluids using a variance-reduced, stochastic implementation of a “micro-macro” method [38]. Further study of multiphase non-Newtonian flows was carried out by Bajaj et al. [39] and Xu et al. [40] using the Brownian Configuration Field (BCF) method of Hulsen et al. [41].

At present, there is a growing interest in Machine Learning (ML) [42,43] in the context of polymer simulation: Doblies et al. [44] employed ML as a means of predicting mechanical properties, while Jackson and collaborators [45] focused on the optoelectronic properties of conjugated polymers. It was also used by Kopal and co-workers in the prediction of the viscoelastic behavior of elastomer systems [46] using Radial Basis Functions (RBFs) or as a data-driven classification method to determine polymer/solvent compatibility by Chandrasekaran et al. [47]. Fluid dynamics of multiphase flow also benefits from this recent focus on ML: Ma and collaborators used statistical learning in [48] for bubbly systems; Ladický et al. accelerated multiphase simulations in GPUs using a data-driven approach with regression forests [49]; and Gibou and co-workers employed deep learning techniques with sharp interface methods in [50]. Hence, it seems unquestionable that ML and data-driven simulations are already having a tremendous impact within the scientific community, and the future possibilities seem nearly endless.

The main purpose of this paper is to highlight the effects caused by the non-Newtonian behavior of the polymer solution in a multiphase flow system, performing a series of numerical simulations by means of a computationally efficient, accurate, and robust numerical method based on a finite element discretization of the governing equations that uses ML-inspired techniques for the reconstruction of the polymer stress tensor, a feature that proves to be of the greatest importance for the accurate characterization of the viscoelastic flow. Thus, after presenting this Introduction, we describe the governing equations along with the aspects of the computational implementation in the Materials and Methods Section 2. Then, we move to Section 3, where we present results for drop deformation under steady, shear flows and in situations where gravitational effects drive the dynamics of the flow. Finally, Section 4 offers some conclusions and future lines of work.

## 2. Materials and Methods

In this section, we describe the main ideas, mathematical background, and computational implementation of the FEM-based, ML-enhanced method used to perform the series of numerical experiments carried out in Section 3 to gain insight into the impact that viscoelasticity may have on drop deformation. We start with the finite element discretization, then moving on to the computational implementation.

### 2.1. Finite Element Discretization

The problem of drop deformation in polymer solutions can be tackled using different methods. In this work, we use an FEM-based discretization of the “macro-scale” equations governing the fluid flow, employing a stochastic modeling of the stresses arising from the polymer fluid to account for the “micro”-scale. Finally, we use a Particle Level Set (PLS) method to capture the interface of the deforming drop.

#### 2.1.1. Governing Equations

The two-phase, incompressible fluid flow studied in this work is governed, at the “macro”-scale, by the Navier–Stokes equations, which in dimensionless form can be written in the space-time domain Ω×[0,T] as:

(1) $$\left\{\begin{array}{l} Re\rho \frac{Du}{Dt}-\nabla \cdot \left[\mu \left(\nabla u+{\left(\nabla u\right)}^{T}\right)\right]+\nabla p=-\rho e_{z}\frac{Re}{Fr^{2}}+\frac{c}{De}\nabla \cdot \tau_{p}+\frac{Re}{We}\kappa \delta_{\Gamma }\left(\phi \right)n,\\ \nabla \cdot u=0;\\ u\left(x,0\right)=u_{0}\left(x\right)\;\forall x\in \Omega ,\\ u\left(x,t\right)=0\;\mathrm{on}\;\delta D_{\mathrm{no}-\mathrm{slip}}\subset \delta \Omega ,\forall t\in \left(0,T\right),\\ u\left(x_{\mathrm{img}},t\right)=\left(x_{\mathrm{ref}},t\right)\;\mathrm{on}\;\delta D_{\mathrm{PBC}}\subset \delta \Omega ,\forall t\in \left(0,T\right),\\ u\left(x,t\right)\cdot n=0\;\mathrm{and}\;n\cdot \tau_{s}\cdot t=0\;\mathrm{on}\;\delta D_{\mathrm{free}-\mathrm{slip}}=\delta \Omega /\left(\delta D_{\mathrm{no}-\mathrm{slip}}\cup \delta D_{\mathrm{pbc}}\right),\forall t\in \left(0,T\right). \end{array}\right.$$

where ρ is the density, μ the viscosity, D()/Dt the total (convective) derivative operator, *p* the pressure, v the velocity, $\tau_{p}$ the polymer (“extra-”)stress tensor, Γ the interface of the drop, κ its curvature, ϕ the level set function (see Section 2.1.2), Re the Reynolds number, Fr the Froude number, We the Weber number, *c* the concentration parameter, De the Deborah number, *T* the final time, and δΩ the boundary of the spatial domain Ω. The “no-slip”, “free-slip”, and “Periodic Boundary Conditions” (PBC), are implemented in Section 2.2.3 for the configurations explored in the Results and Discussion Section 3. Using a semi-Lagrangian approach, the total convective operator in Equation (1) is discretized along the “characteristic curves” of the flow without changing the Eulerian, underlying mesh. The resulting weak formulation can be written as a Stokes-like problem to be solved at each instant of time, with the following form:

(2) $$\left\{\begin{array}{c} \frac{3Re}{2dt}\left(\rho^{*}\left(\phi_{h}^{n}\right)u_{h}^{n},\psi_{h}\right)+\left(\mu^{*}\left(\phi_{h}^{n}\right)\nabla u_{h}^{n},\nabla \psi_{h}\right)-\left(p_{h}^{n},\nabla \cdot \psi_{h}\right)=\frac{2Re}{dt}\left(\rho^{*}\left(\phi_{h}^{n}\right){\bar{u}}_{h}^{n-1},\psi_{h}\right)-\\ -\frac{Re}{2dt}\left(\rho^{*}\left(\phi_{h}^{n}\right){\bar{u}}_{h}^{n-2},\psi_{h}\right)-\frac{Re}{Fr^{2}}\left(\rho^{*}\left(\phi_{h}^{n}\right)e_{z},\psi_{h}\right)+\\ +\frac{c}{De}\left(\nabla \cdot \tau_{ph}^{n},\psi_{h}\right)+\frac{Re}{We}\left(\kappa_{h}^{n}\delta_{\Gamma_{h/2}}\left(\phi_{h}^{n}\right)n_{h}^{n},\psi_{h}\right),\forall \psi_{h}\in V_{h0};\\ \left(\nabla \cdot u_{h}^{n},q_{h}\right)=0,\;\forall q_{h}\in Q_{h};\;\mathrm{with}:\left(a,b\right)\equiv \int_{D}ab\;dx \end{array}\right.$$

where the subscript ${}_{h}$ denotes the spatial discretization of the variables; the superscripts ${}^{*}$ and ${}^{n}$ their non-dimensionality and temporal discretization at instant of time *n*, respectively; dt is the time step size used in the discretization of the full interval (0,T); $\psi_{h}$ the basis functions of velocity vector space $V_{h}$ and $q_{h}$ the basis functions of the pressure space $Q_{h}$. The basis functions of the finite element spaces are chosen to be polynomials of degree $\left\{P_{2},P_{1},P_{2},P_{1}\right\}$ for velocity, pressure, the level set function, and the polymer stress tensor, respectively, thus satisfying the Ladyzhenskaya–Babus̆ka–Brezzi condition. The spatial discretization of the domain Ω is accomplished using an unstructured mesh composed of simplices of average size *h*, and the surface tension effects are implemented making use of the Laplace–Beltrami operator, as detailed in [37].

In Equation (2), we collect the “macro-scale” physics involved in the FEM-based discretization of a multiphase flow of incompressible fluids subject to gravity, viscous, and surface tension forces, in which the interface is retrieved by a level set function ϕ, and the viscoelastic effects of the polymer solution are captured through the polymer stress tensor $\tau_{p}$. We now take a stochastic approach to the computation of $\tau_{p}$ avoiding closed-form constitutive equations, performing instead Brownian dynamics simulations of polymer particles [7] modeled via the Hooke (equivalent to the Oldroyd-B constitutive equation) and FENE (Finitely Extensible Non-linear Elastic) kinetic models [51]. To compute $\tau_{p}$, we need to average over the internal configurations Q of each of the $N_{d}$ dumbbells (“polymer particles”), which are scattered over the domain and follow the flow. Thus, we solve for each of the dumbbells the following stochastic (dimensionless) differential equation:

(3) $$dQ=\left\{\begin{array}{cc} \left(\kappa \cdot Q-\frac{1}{2De}Q\right)dt+\frac{1}{\sqrt{De}}dW, & \mathrm{Hooke};\\ \left(\kappa \cdot Q-\frac{1}{2De}\frac{Q}{1-{∥Q∥}^{2}/b}\right)dt+\frac{1}{\sqrt{De}}dW, & \mathrm{FENE}; \end{array}\right.$$

a task that is accomplished by means of a (weak) second-order accurate predictor-corrector algorithm proposed by Öttinger [51]. In Equation (3), κ is the velocity gradient, W a stochastic Wiener process, the Deborah number De representing the ratio between the relaxation time of the polymer and a characteristic time of the flow, and *b* is the FENE extensibility parameter so that, as b→∞, Hooke→FENE. A variance-reduction technique can also be implemented [37,41], performing the average over the $N_{d}$ dumbbells with uncorrelated noise that comprise each of the $N_{ens}$ ensembles, so that the *i*-th dumbbell of each ensemble is subject to the same (correlated) stochastic “kick”, reducing the overall noise of the simulation. Finally, the polymer stress tensor is reconstructed and evaluated at the mesh nodes [52] using the ML-inspired techniques of Section 2.2.1.

#### 2.1.2. Interface Capturing Technique

The interface Γ is captured as the zero isocontour of a level set function ϕ, which is transported by the characteristic curves of the flow through the total derivative operator:

(4) $$\frac{D\phi }{Dt}=\frac{\partial \phi }{\partial t}+v\cdot \nabla \phi =0.$$

As we did with Equation (1), the convective terms in Equation (4) are dealt with using a semi-Lagrangian approach, as proposed first in [53]. The level set function ϕ is initialized as a signed-distance function to improve the regularity and behavior of the transported solution. However, as time goes on, discretization errors build up in the form of numerical artifacts that may eventually propagate into the interface and destroy it. To prevent this undesired effect, an Eikonal-based, redistancing procedure [54] is performed at each time step, so that we ensure that, within a band around the interface, the level set function preserves the signed-distance property. To improve mass conservation and shape definition, we add $N_{mp}$ marker particles [18] that are passively advected by the flow, helping to correct the interface by defining local level set functions with a variable radius $0\leq r_{\min}\leq r_{p}^{n}\leq r_{\max}<h$, accounting for lost resolution in the sub-grid scales (see [37] for details).

### 2.2. Computational Implementation

Next, we describe some of the topics involved in the computational implementation of the ML-enhanced, FEM-based method utilized to tackle the drop deformation problems proposed in Section 3. All code was written in the C programming language and compiled using the open-source GNU-C compiler gcc. Efficiency and robustness are two features pursued in this implementation; accordingly, all simulations presented in this work can be run in a commodity personal computer with a 4-core processor. All 2D plots were made using the open-source library matplotlib [55] and/or the Ti*k*Z and PGF packages [56], while the 3D graphics make use of Mayavi [57]. For data manipulation and data analysis, we use the pandas [58] package.

#### 2.2.1. Machine Learning Enhancement

Radial basis functions are a family of kernel methods [59] widely used in Machine Learning (ML) for their flexibility in generating spaces of trial functions with excellent approximation properties. This close relation to approximation theory allows for a solid mathematical background, which proves extremely useful when offering bounds in supervised learning problems [43] for the necessary number of training data required to provide a model trained with such data and having a small generalization error. Combining these concepts with novel applications in the field of image processing [60,61], we leverage RBFs to reconstruct the discrete solution of the polymer stress tensor over the whole domain, using the scattered data provided by the values of the polymer stress tensor defined at each of the position of the ensembles. The main advantage of this idea is the mesh independence property guaranteed by the RBF approach; the relevance of this feature cannot be overstated in the context of “micro-macro” methods for non-Newtonian flows [29,34,41]. Traditional methods use particles for the kinetic modeling of the polymer solution; however, spatial mesh refinement hinders the accuracy of the stochastic model (given the reduced number of particles surrounding a certain mesh node), thus requiring compromising on the accuracy of either the “micro-” (internal configurations of the polymer and “extra-”stress tensor) or the “macro-” (velocity, pressure) scales. This is no longer the case with our approach: now, using the smooth approximation built via RBFs, we can evaluate the tensor at any position (e.g., a certain mesh point), virtually decoupling the “micro-” and “macro-”scales and paving the way for adaptive mesh refinement in “micro-macro” methods.

Thus, at each instant of time, we retrieve the positions $\left\{x_{i}\right\},i=1,..,N_{ens}$ of all the ensembles scattered over the domain. Then, we compute $\tau_{p,i}$, the value of the polymer stress tensor at the ensemble *i* using Kramers’ expression averaging over the $N_{d}$ dumbbells contained at the ensembles:

(5) $$\tau_{p,i}=nk_{B}\Theta \frac{1}{N_{d}}\sum_{j=1}^{N_{d}}F(Q_{j}^{i})⊗Q_{j}^{i}-I.$$

As kernels of approximation, we focus on compactly-supported RBFs [59,62] for their computational efficiency and remarkable accuracy, since the matrices resulting from a suitable formulation of the approximation problem are sparse. In particular, we use Wendland’s $\varphi_{3,k}\left(C^{2k}\right),k=0,..,3$, suitable for problems of space dimension d≤3, and degree of smoothness 2k:

(6) $$\left\{\begin{array}{cc} \varphi_{3,0}\left(r\right) & ={\left(1-r\right)}_{+}^{2},\\ \varphi_{3,1}\left(r\right) & ={\left(1-r\right)}_{+}^{4}\left(4r+1\right),\\ \varphi_{3,2}\left(r\right) & ={\left(1-r\right)}_{+}^{6}\left(35r^{2}+18r+3\right),\\ \varphi_{3,3}\left(r\right) & ={\left(1-r\right)}_{+}^{8}\left(32r^{3}+25r^{2}+8r+1\right), \end{array}\right.$$

so that outside the support size χ, $\varphi_{3,k}\left(r>\chi \right)=0$, whereas inside the support, they are positive definite on $R^{d}$ and provide optimal convergence rate $O{\left(h_{\Omega }\right)}^{2k+d+1}$, with $h_{\Omega }$ the fill distance [62]. Using these kernels, we build an interpolant *s* of the form:

(7) $$s\left(x\right)=p\left(x\right)+\sum_{i}\lambda_{i}\varphi \left(∥x-x_{i}∥\right),$$

with p(x) a quadratic function defined by coefficients $c_{i}$ and and $\lambda_{i}$ a set of real-valued interpolation coefficients for the CSRBFs. The formulation can then be reduced to a system of equations equivalent to a saddle-point problem of the form [Q P; P^T^ 0]] [l; c] = [f; 0], which can be solved efficiently using the two-step approach described in [52] to obtain the solution vectors [l;c] required to build the interpolant *s*. After building the interpolant and having reconstructed the polymer stress tensor, we can straightforwardly evaluate each component at any given point (e.g., at one mesh node).

The use of CSRBFs as approximation kernels calls for the determination of the support size χ, for each of the ensembles scattered over the domain. The technique used to perform this potentially expensive computation is taken from the classification problems usually found in ML: the Nearest Neighbor (NN) classification algorithm [42,43]. Using this approach, along with an implementation of kd-tree as data structures, we are able to obtain, at each instant of time, the nearest neighbors of each ensemble, within a certain support size χ.

#### 2.2.2. PETSc-Based Solver

The solution of the linear systems arising from the FEM discretization of the coupled velocity-pressure system is carried out taking advantage of PETSc, the Portable, Extensible Toolkit for Scientific Computation [63]. This step is key to the overall efficiency and viability of the method as an appropriate candidate to tackle Newtonian and non-Newtonian multiphase flows in which large density and viscosity ratios are involved, as those taking place in experiments dealing with drop and bubble deformation. As the ratio increases, so does the condition number of the resulting velocity matrix, which hampers the convergence of traditional iterative approaches to the solution of the coupled velocity-pressure system such as preconditioned conjugate gradient techniques [64] or traditional “splitting” techniques, usually problematic at low (viscosity-dominant) Reynolds numbers [65]. Instead, we use a physics-based, block-preconditioning approach presented by Elman and collaborators [66] and implemented in the PETSc-FieldSplit preconditioner: we define separate fields for the discrete velocity and pressure and combine them using a Schur-complement factorization [67] of the total system matrix, which is efficiently stored as a MatNest structure K = [A B; B^T^ 0], in which the corresponding sub-matrices for the discrete velocity, discrete gradient, and discrete divergence are the four entries K_ij, for i, j = 1, 2. The following snippet collects a sample function to build the block matrix.

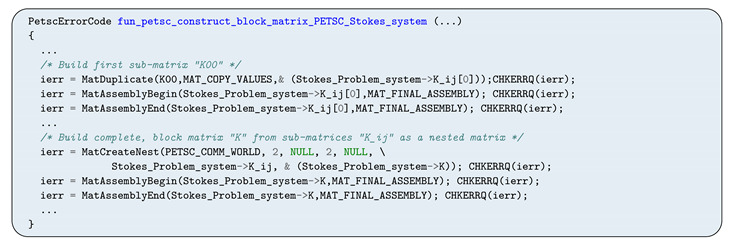



The previous approach can now be used to tackle the saddle-point problem resulting from our FEM-based discretization of the Navier–Stokes equations using the PETSc-FieldSplit preconditioner for the system matrix, collecting all the information in a suitable structure such as the PETSc_Saddle_point_system sample structure included in the following code snippet.

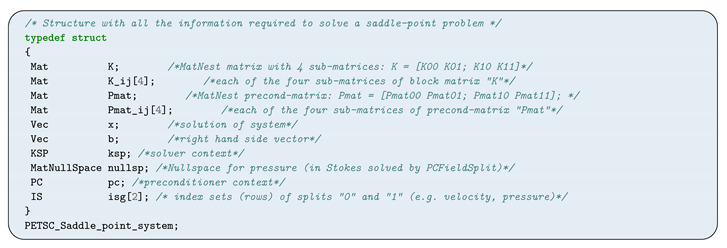



The CHOLMOD package [68] is used to perform the sparse direct Cholesky factorization of the positive-definite block matrix K00=A, while the Least-Squares Commutator (LSC) preconditioner [67] takes care of the second field (pressure) involved in the upper factorization of the Schur complement, using a combination of the conjugate gradient and additive-Schwarz methods [69] for the multigrid levels of the ML (Multi-Level) preconditioner package [70] used for LSC. The following options passed to PETSc were found to offer excellent performance in demanding multiphase flow problems with high density ratios such as those found in Section 3.2.

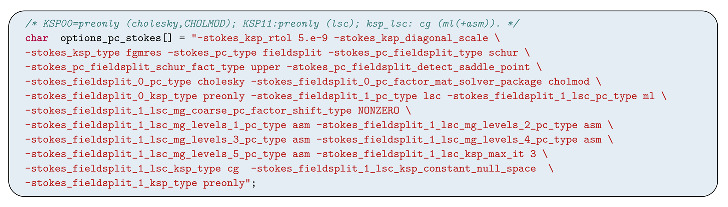



These are some of the general configuration options and data structures that are most responsible for the performance boost we find for the kind of complex, polymer flows studied in this work. Given the platform-independent model of C and PETSc, the snippets could be included with little modification in any existing C code used by any researcher with a similar underlying mathematical problem, independently of the particular field of knowledge.

#### 2.2.3. Boundary Conditions

The viscous and viscoelastic flows presented here for the analysis of drop deformation in polymer solutions make use of several types of boundary conditions: the “no-slip” boundary condition to ensure that the fluid adheres to the adjacent solid boundary; the “free-slip” boundary condition to neglect friction forces at the fluid-solid interface; and Periodic Boundary Conditions (PBCs) at the inlet and outlet of the domain to model sufficiently large domains.

**Figure 1:**
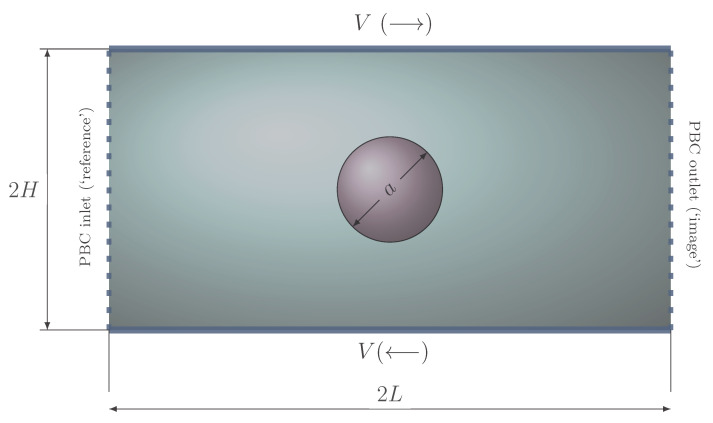
Schematic of the computational domain for a drop deforming in shear flow.

In our FEM-based method, the “free-slip” condition can be applied naturally as a Neumann-type condition that is satisfied automatically by the weak formulation of the problem [7,37]. However, the implementation of PBCs in unstructured meshes for the configuration presented in Section 3.1 for drop deformation in shear flows is not straightforward. Following Pask et al. [71] and Sukumar and Pask [72], we carry out an efficient implementation of the PBCs in structured and unstructured finite element meshes using the idea of “reference” and “image” nodes at the inlet and outlet, respectively (see Figure 1). First, we obtain the connectivity lists for the assembled sub-matrices K_00,K_01,K_10 of the block matrix K = [K00 K01; K10 K11]; then, we perform row operations (first) and column operations (later) to K00,K01,K10 and to the right-hand side F, as described in [72]; finally, we update the values of the (pressure, velocity) “image” nodes with the values obtained from the corresponding “reference” nodes. The addition of dumbbells and marker particles to deal with multiphase flows and non-Newtonian fluids adds complexity to the actual implementation, but not to the general concept.

As for the “no-slip” conditions, they translate into homogeneous or non-homogeneous Dirichlet-type conditions that can be applied efficiently using the symmetry-conserving procedure MatZeroRowsColumns to perform row and column operations on the PETSc-based assembled matrices K00,K01,K10 and right-hand side F.

## 3. Results and Discussion

The main purpose of this paper is to explore the behavior of drops immersed in polymer solutions through a series of numerical experiments that try to highlight the effects of viscoelasticity in the shape of the drop, as well as in the emerging flow patterns. First, we focus on drop deformation under a steady, shear flow, offering results under two configurations: Newtonian drop in a Newtonian “matrix” (ambient fluid) and non-Newtonian drop in a Newtonian matrix. Then, we make use of our ML-enhanced, FEM-based method to investigate stronger viscoelasticity effects in flows where gravitational forces and surface tension effects can become relevant, showing the influence that the discretization employed and the numerical techniques utilized may have on the accurate reconstruction of the polymer stress tensor and on the quality of the discrete solution for the multiphase flow.

### 3.1. Drop Deformation in Steady, Shear Flow

We start the exploration of viscoelastic effects on drop deformation by considering the problem of a drop immersed in another, ambient fluid and subject to a steady, shear flow. First, we show results for a Newtonian/Newtonian system; then, we introduce viscoelasticity in the system, comparing our results with others obtained using a constitutive (Oldroyd-B) model, equivalent to our stochastic, Hookean dumbbell approach. The configuration is represented in Figure 1: a drop of a viscous (real) fluid of radius *a* placed at the center of a domain [2L×2H], with H=4a and L=8a, experiences a steady, shear flow of rate $\dot{\gamma }=V/H$ produced by the top and bottom lids moving at velocity *V* in opposite directions.

The inlet and outlet walls have Periodic Boundary Conditions (PBCs), the implementation of which is detailed in Section 2.2.3. At the top and bottom panels, the “no-slip” boundary condition is considered.

#### 3.1.1. Newtonian Drop in a Newtonian Matrix

**Figure 2:**
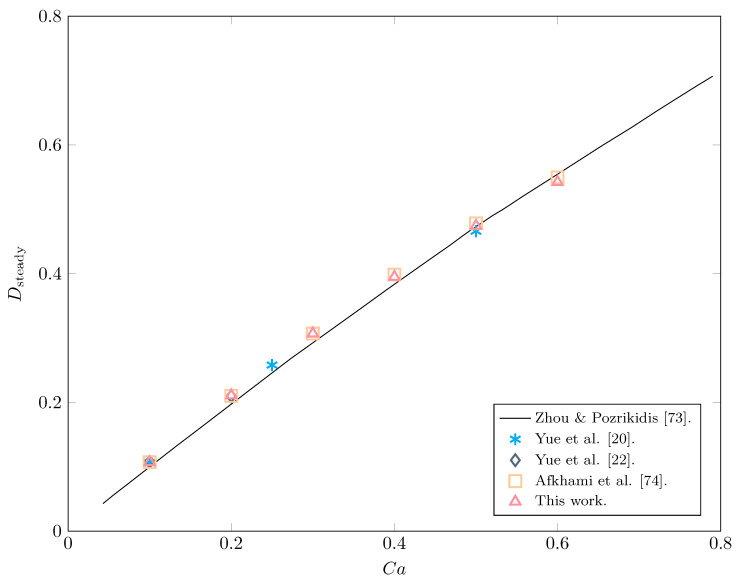
Comparison of the steady-state deformation of a Newtonian drop in a Newtonian matrix between our results and those found in Zhou & Pozrikidis [73], Yue et al. [20,22], and Afkhami et al. [74].

We place a Newtonian drop in a Newtonian ambient fluid as depicted in Figure 1 to study the shape of the interface for different values of the capillary number Ca=We/Re in terms of the “deformation parameter” *D*. This parameter is usually defined as $D=\left(L-B\right)/\left(L+B\right)$, with *L* and *B* being the longest and shortest lengths from the center of the drop to the interface (corresponding also to the major and minor axes of the ellipse), respectively. We also choose a small value for the Reynolds number (Re=0.1) so as to neglect strong inertial effects, comparing our results with those of Zhou and Pozrikidis [73], Yue et al. [20,22], and Afkhami et al. [74]. We employ an unstructured, uniform mesh of size h=1/80, in a rectangular domain [0,2]×[0,1] with time step dt=1/100 and $N_{mp}=2.5\times 10^{5}$ marker particles. In Figure 2, we represent the steady value of the deformation parameter for increasing values of the capillarity number; in all cases, we obtain excellent agreement with the published results, especially with that by Afkhami et al. [74], deviating in the worst case scenario <2.5% at Ca=0.6 from the results offered by Zhou and Pozrikidis in [73]. The steady values of *D* show a linear behavior with Ca for small capillary numbers, then decreasing as Ca gets larger; as pointed out in [20], we expect the physical insights gained by this 2D exploration to be relevant to actual (three-dimensional) experiments.

**Figure 3:**
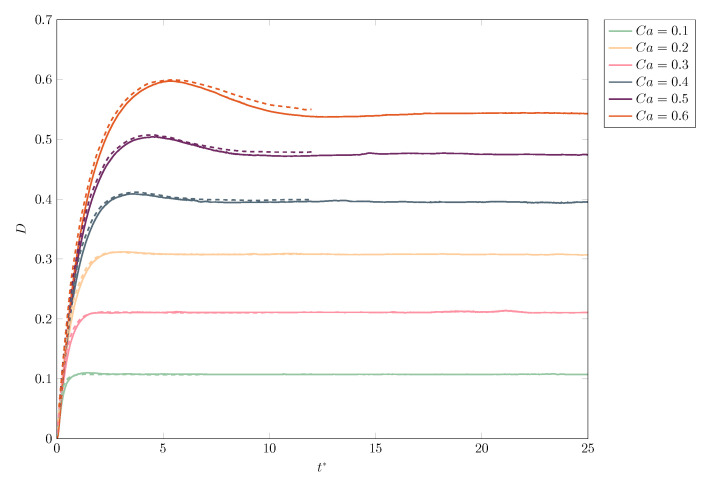
Comparison of the evolution of deformation for a Newtonian drop in a Newtonian matrix of equal viscosity, for different values of capillarity Ca, between our results (solid line) and those found in Afkhami et al. [74] (dashed line).

We also collect in Figure 3 the evolution of the deformation parameter *D* as a function of the dimensionless time $t^{*}=t\dot{\gamma }$ and compare it with the results of [74], for a wide range of capillary numbers from Ca=0.1 to Ca=0.6. Again, an excellent agreement is obtained throughout, with a maximum difference of less than 1.3% (for Ca=0.6) between the two numerical methods at any given time.

**Figure 4:**
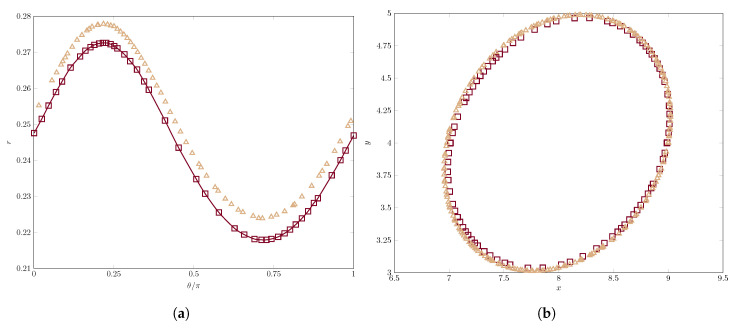
Comparison of the drop shape in (**a**) polar coordinates and (**b**) the actual shape, for *Ca* = 0.1, between our results (triangles) and those found in Yue et al. [22] (square markers).

Following Yue et al. [22], we now study the steady shape of the drop in polar coordinates, collecting the radius *r* for each orientation angle θ∈[0,π], with θ defined as the angle formed by the line connecting the center of gravity of the drop and a point in the free-surface, with the *x*-axis. Thus, we show in Figure 4 the shape in polar coordinates and the actual shape for a simulation with Ca=0.1.

We can observe in this Figure 4 very good agreement in the orientation angle of the droplet, which can be measured at $\hat{\theta }\approx 39.6^{\circ }$, lower than $45^{\circ }$ as numerical and experimental results confirm. However, there is a major discrepancy between the results of the two methods: the radii for each polar angle are smaller for the method of Yue et al. [22] when compared with ours (see left panel). To better understand what is happening here, we plot the actual shape of the drop according to both methods in the right panel of Figure 4. As can be observed, the reason for the discrepancy lies in the lack of mass conservation “mass-loss” incurred by the drop of [22], which loses around 5% of its initial mass at the end of the simulation, whereas we can ensure mass conservation up to 99.98% of the initial mass in this simulation.

#### 3.1.2. Viscoelastic Drop in a Newtonian Matrix

After exploring the multiphase Newtonian/Newtonian system and comparing our results with those of the literature, we now proceed with a problem in which the viscoelastic effects are present. We focus on a viscoelastic drop modeled by the Hookean dumbbell model (equivalent to the Oldroyd-B constitutive equation), immersed in a Newtonian, ambient fluid, using the same configuration of the previous Newtonian/Newtonian analysis. The flow is impulsively started at t=0 with shear rate $\dot{\gamma }=1$, using a rather coarse mesh with 80×40 elements and $N_{p}=10^{7}$ dumbbells uniformly placed inside the droplet; the flow is continued until dimensionless time $t^{*}=t\dot{\gamma }=10$, with a small time step to accurately solve the internal configurations of the dumbbells (dt=1/200), taking $N_{t}=2000$ time steps to finish each simulation; the number of marker particles to improve the definition of the interface was $N_{mp}=2.5\cdot 10^{5}$. The ratio between viscous and inertial effects in the system, represented by the Reynolds number, are of utmost importance, in the sense that, if the method is not able to deal with extremely low Re (“creeping” or “Stokes” flows), the inertial effects become relevant when small time steps are used, thus affecting the history of the flow and, consequently, that of the polymer particles (dumbbells); it is for this reason that the value of Re=0.1, deemed appropriate in the previous section for Newtonian flows, is now replaced by $Re=10^{-5}$ to suppress any undesired effects that inertia may have on the computation of the steady-state values for the polymer solution. The remaining dimensionless parameters are chosen as those found in [21], with Fr→∞, $We=10^{-6}$, so that the Capillary number Ca=0.1; our concentration parameter *c*, according to the characteristic scales chosen in [21], corresponds to c=1−β, with β=0.5 the retardation parameter of the Oldroyd-B fluid; the Deborah numbers studied are $De=\left\{0.25;0.5;1;2\right\}$; and the density and viscosity ratios between the drop and the outer ambient fluid (matrix) are taken as $\rho_{2}/\rho_{1}=\mu_{2}/\mu_{1}=1$. We performed a set of simulations to obtain the evolution of the drop deformation and compared the results found in Figure 1 of [21].

**Figure 5:**
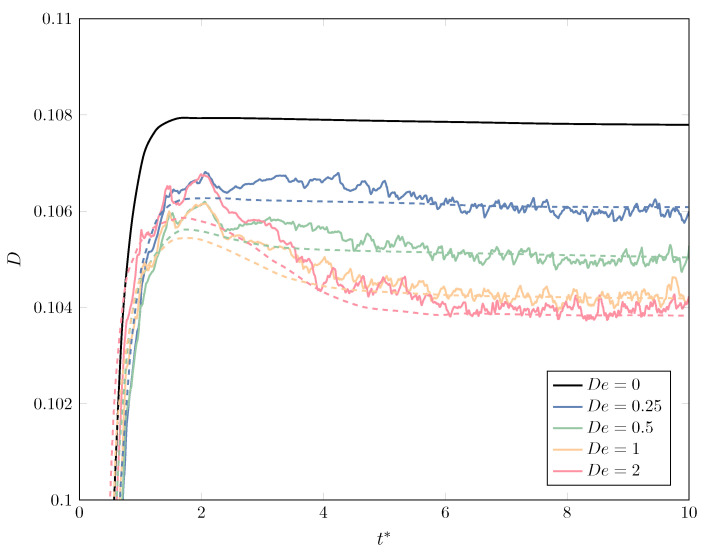
Comparison of the evolution of deformation for a non-Newtonian (Oldroyd-B) drop in a Newtonian matrix of equal viscosity after the abrupt start of shear flow, for increasing values of the Deborah number De, between our results (solid line) and those found in Yue et al. [21] (dashed line).

Despite the rather coarse mesh used (equivalent to a uniform size h=1/40), the not-so high number of dumbbells and the totally different approach taken by the two techniques compared—the diffuse-interface method combined with a phase-field approach ruled by Cahn–Hilliard dynamics to study the Newtonian/non-Newtonian problem in a unified way of [21] and our FEM-based, stochastic method—the results presented in Figure 5 are in remarkably good agreement, especially the steady-state values of the deformation parameter *D*. However, also the transient behavior shows a noteworthy resemblance, with the overshoot appearing for sufficiently high De values and the evolution of De=2 being for t⪅4 higher than those for De=1. In any case, we notice the effect of the drop viscoelasticity as a means to reduce the deformation of the interface; plots of the actual shape of the interface (not included here for brevity’s sake) show this same trend. Apart from the stochastic noise, the modification of the interface by the correction step of the marker particles and the mass conservation step add to the oscillatory behavior of *D* observed in Figure 5, which is explicitly computed from the discrete interface. Moreover, Figure 5 underscores the elastic effects of the drop, since an increase in the Deborah number De produces a larger ratio between the maximum and the steady-state value of the deformation parameter *D*, as a consequence of the longer time (slower response) that the polymer molecules take to recover from the applied shear strain.

**Figure 6:**
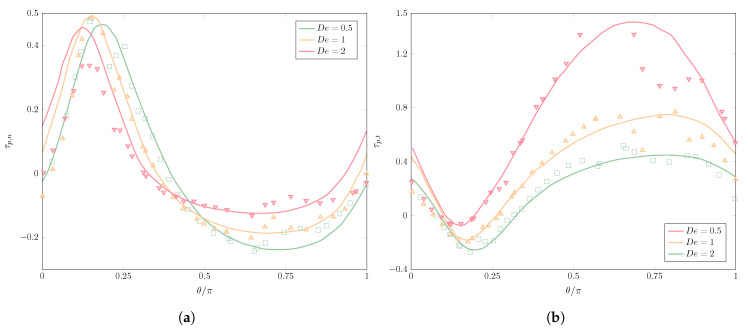
Comparison of the (a) normal and (b) tangential stresses along the inner edge of the interface for a non-Newtonian (Oldroyd-B) drop in a Newtonian matrix of equal viscosity, for increasing values of the Deborah number, between our results (*De* = 0.5, green squares; *De* = 1, yellow triangles; *De* = 2, inverted red triangles) and those found in Yue et al. [22].

We now compare the steady-state values of the polymer stress tensor obtained using the Oldroyd-B constitutive model of [22] and the Hookean dumbbell model. The results are presented in Figure 6. As we did for the Newtonian/Newtonian case, we represent the interface also in polar coordinates with θ∈[0,π]. The normal component of the polymer stress tensor $\tau_{p,n}\equiv n\cdot \tau_{p}\cdot n$ (with n the outer normal at each point of the interface) is computed in Figure 6a.

In spite of the largely dissimilar methods, we notice a very good agreement between them. We observe how the effects of the viscoelastic drop are not very strong on the normal component of the polymer stress tensor, though increasing the viscoelasticity by means of De reduces the maximum value, which is also moved towards smaller orientation angles, thus playing a part in diminishing drop deformation. Overall, the normal polymer stress component is weakened at the poles and at the equator of the drop as viscoelasticity increases, preventing drop deformation.

Figure 6b shows the tangential component of the polymer stress tensor $\tau_{p,t}\equiv t\cdot \tau_{p}\cdot t$, with t the unit vector tangent at all points to the interface and perpendicular to n (so that, in 2D, $t_{x}=n_{y},t_{y}=-n_{x}$). Here, the polymer stresses are much larger than for the normal component, showing again an excellent agreement between our results and those in [22]. The larger De values intensify the tangential stresses at the equator, being responsible to a larger extent for the reduced deformation observed for the higher De. Despite small discrepancies, even with a coarse mesh and computationally inexpensive simulation, our results compare very well with those of the reference, capturing the minimum and maximum values of $\tau_{p,t}$, as well as the evolution of $\tau_{p,t}$ along the interface.

### 3.2. Drop Deformation in Buoyancy-Driven Flow

After studying the problem of drop deformation in unsteady, shear flow, in this section, we focus on following the behavior of a Newtonian drop immersed in an ambient, polymeric solution, rising due to buoyancy effects and the density ratio between the fluids [75]. By means of a series of numerical experiments, we gain insight into the deformation of the drop shape, characterized by the circularity c, the ratio between the perimeter of a circle whose area is equal to that of the drop and the perimeter of the drop. We also shed light on the response of the “extra-” stress tensor $\tau_{p}$ to a varying degree of polymer concentration and relaxation time, when the drop undergoes large deformation and velocity gradients, highlighting the influence of different smoothness in the CSRBF. Finally, we observe the effects that viscoelasticity may have on the flow, with emphasis on the flow streamlines during the full, unsteady simulation.

Thus, we consider a dimensionless, 2D rectangular domain [0,1]×[0,2] in which a drop of radius R=0.25 is placed at position (0.5,0.5) inside an unstructured mesh of uniform size *h*. The outer, non-Newtonian polymer solution is modeled using the FENE and Hookean dumbbell models. The flow is further defined by the density and viscosity ratios $\rho_{2}/\rho_{1},\mu_{2}/\mu_{1}$ and dimensionless numbers Re,We,Fr,c,De. The “no-slip” boundary condition is applied at the top and at the bottom, while we consider “free-slip” at the lateral boundaries. All simulations are carried until dimensionless time t=3.

#### 3.2.1. Convergence Results

We turn our attention to the evolution of the drop shape during the full simulation, as the mesh size is decreased from h=1/40 down to h=1/320, and the number of ensembles scattered over the domain changes, using two different kinetic models, Hooke, and FENE, and different degrees of viscoelasticity, (c=1,De=1) and (c=5,De=3). The other relevant parameters in this case are: $Re=35,We=10,Fr=1,\rho_{2}/\rho_{1}=10^{-1}=\mu_{2}/\mu_{1}$.

**Figure 7:**
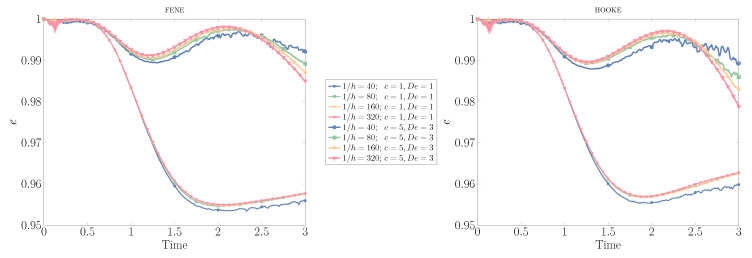
Evolution of the circularity of a drop rising due to buoyancy effects in a polymer solution, using the FENE and Hookean kinetic models, variable mesh size $h=\left\{1/40,1/80,1/160,1/320\right\}$, and different degrees of viscoelasticity: c=1,De=1; and c=5,De=3.

In Figure 7, we present the results for convergence under mesh refinement, for the FENE and Hooke models: for the case with weaker viscoelastic effects (c=1,De=1), the number of ensembles is $N_{ens}=5000$, while the number of dumbbells inside each ensemble is $N_{d}=150,000$ for both stochastic models. When the polymer concentration is increased (c=5,De=3), we scatter more ensembles in the ambient (polymeric) fluid $N_{ens}=5000$ to capture the more dramatic effects in shape deformation, reducing the number of dumbbells within each ensemble to $N_{d}=15,000$ to keep the computational demands at a similar level. The results obtained here compare very well with those provided in [7] for purely uncorrelated dumbbells, showing excellent convergence with mesh refinement for both kinetic models and degrees of polymer concentration. Only in the coarser mesh, h=1/40, do we observe a slightly different behavior to that produced by the other unstructured meshes. This pattern is more evident when using the Hooke instead of the FENE dumbbell model, due to the larger stresses produced by the former model during drop deformation: the minimum value of the circularity decreases for both c=1 and c=5, and we observe a lower local maximum at t≊2.2 for c=5,De=3, with the drop showing a lack of deformation at the latter stages of the simulation (t≥2.75).

**Figure 8:**
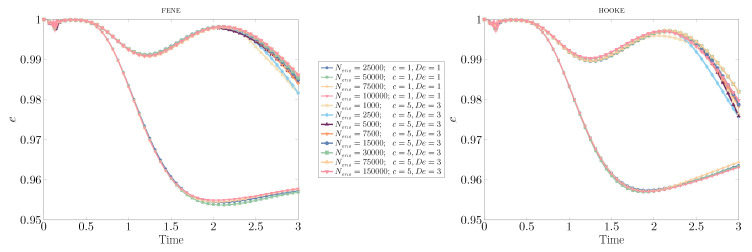
Evolution of the circularity of a drop rising due to buoyancy effects in a polymer solution, using the FENE and Hookean kinetic models, variable number of ensembles ($N_{ens}\times N_{d}=750\times 10^{6}$), and different degrees of viscoelasticity: c=1,De=1; and c=5,De=3.

Figure 8 shows the evolution of the circularity when the number of ensembles $N_{ens}$ and dumbbells per ensemble $N_{d}$ changes, keeping the product $N_{ens}\times N_{d}=750\times 10^{6}$ constant so as to maintain the computational cost; the mesh size is h=1/320. For the case with lower polymer concentration and viscoelastic effects, the convergence results are remarkable for both models. When the concentration parameter and Deborah number are increased to c=5,De=3, we observe a small discrepancy in the circularity values, proving that a certain number of ensembles are required to reconstruct, in an accurate way, the stronger polymer stresses. However, there seems to be an optimal value of $N_{ens}$ and $N_{d}$ for a given computational cost, with this value of $N_{ens}$ being larger for Hooke than for the FENE model. In any case, such a modest diverging behavior is only observed at or after the minimum circularity (c=1) or local maximum circularity (c=5) are attained.

#### 3.2.2. Impact of CSRBF smoothness on the polymer stress tensor

Next, we investigate the influence that the degree of CSRBF smoothness has on the flow. In particular, we are interested in observing qualitative and quantitative deviations in the shear component and normal stress difference of the polymer stress tensor $\tau_{p}$, in a case with strong viscoelastic effects (c=5,De=3), when Wendland’s CSRBFs, $\varphi_{3,k},\forall j=0,..,3$, are used. The reason behind our pursuing this investigation is twofold: since the “extra-”stress is responsible for the viscoelastic effects, it is mandatory to represent it as accurately as possible in simulations that aim to highlight the non-Newtonian behavior of the polymer solution; at the same time, this should be accomplished as efficiently as possible, opting for the CSRBFs that provide results nearly as accurate as the more computationally expensive alternatives.

**Figure 9:**
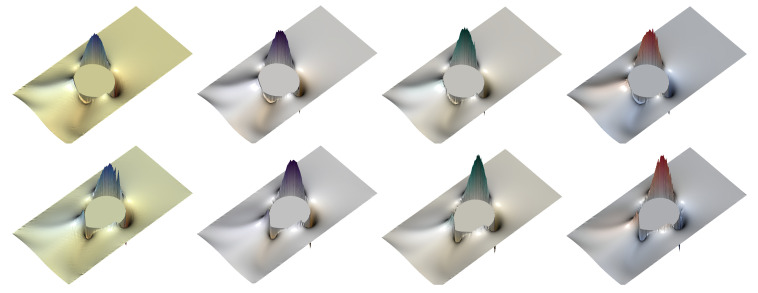
Representation of the shear component of the polymer stress tensor, $\tau_{p,12}$, for the FENE model (b=50) with c=5,De=3, at dimensionless instants of time t=2 and t=3, for variable types of CSRBF. From left to right: $\varphi_{3,0},\varphi_{3,1},\varphi_{3,2},\varphi_{3,3}$. Top row: t=2; bottom row: t=3.

**Figure 10:**
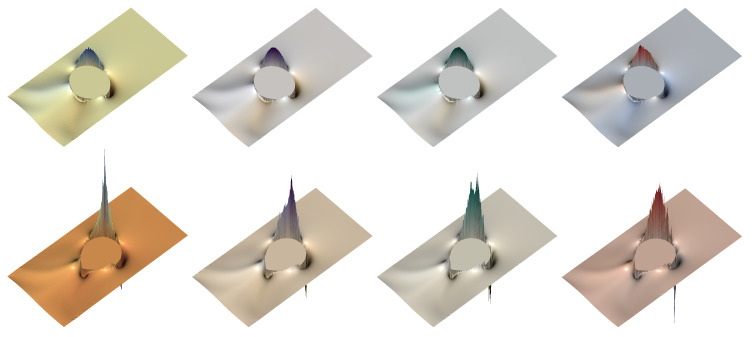
Representation of the shear component of the polymer stress tensor, $\tau_{p,12}$, for the Hookean dumbbell model (Oldroyd-B) with c=5,De=3, at dimensionless instants of time t=2 and t=3, for variable types of CSRBF. From left to right: $\varphi_{3,0},\varphi_{3,1},\varphi_{3,2},\varphi_{3,3}$. Top row: t=2; bottom row: t=3.

**Figure 11:**
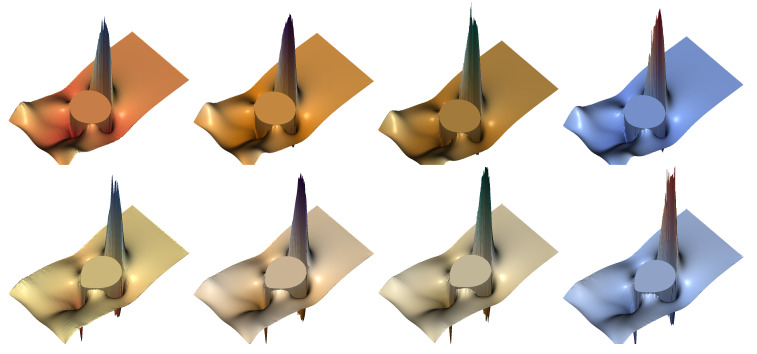
Representation of the normal stress difference of the polymer stress tensor, $\tau_{p,11}-\tau_{p,22}$, for the FENE model (b=50) with c=5,De=3, at dimensionless instants of time t=2 and t=3, for variable types of CSRBF. From left to right: $\varphi_{3,0},\varphi_{3,1},\varphi_{3,2},\varphi_{3,3}$. Top row: t=2; bottom row: t=3.

**Figure 12:**
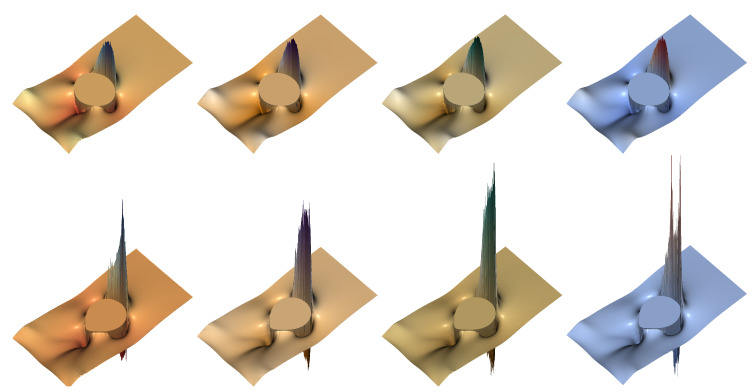
Representation of the normal stress difference of the polymer stress tensor, $\tau_{p,11}-\tau_{p,22}$, for the Hookean dumbbell model (Oldroyd-B) with c=5,De=3, at dimensionless instants of time t=2 and t=3, for variable types of CSRBF. From left to right: $\varphi_{3,0},\varphi_{3,1},\varphi_{3,2},\varphi_{3,3}$. Top row: t=2; bottom row: t=3.

In the following simulations, we take an unstructured mesh of size h=1/320, keeping the same values of the dimensionless parameters found in the previous section. The number of ensembles for the FENE model is $N_{ens}=30,000$, and the number of dumbbells within each ensemble $N_{d}=25,000$; for the numerical experiments using the Hookean dumbbell model, the parameters are $N_{ens}=75,000$ and $N_{d}=10,000$. Thus, Figure 9, Figure 10, Figure 11 and Figure 12 show the shear component $\tau_{p,12}$ and the normal stress difference $\tau_{p,11}-\tau_{p,22}$, at (dimensionless) instants of time t=2 and t=3; for the ease of representation, the Hookean stresses are scaled down a factor of 1.81 from the results obtained with the FENE model.

For the shear component $\tau_{p,12}$ of the polymer stress tensor using the FENE model (Figure 9), all CSRBFs provide very smooth solutions at t=2. At the final time of the simulation t=3, when larger stresses are produced, the Wendland $\varphi_{3,0}$ CSRBF shows small oscillations and a certain roughness that may translate into an insufficiently resolved velocity field and an overall loss of symmetry in the drop shape. Wendland $\varphi_{3,1}$ offers much better accuracy, as well as an improved spatial resolution of the stresses, to such an extent that increasing the smoothness of the CSRBF ($\varphi_{3,0}$, $\varphi_{3,3}$) offers almost no distinct enhancement.

When the Hookean dumbbell model is used (Figure 10), $\tau_{p,12}$ shows much larger values throughout the whole simulation. The influence of the type of CSRBF becomes evident at the later stages, when extreme and very localized stresses are observed. The less smooth $\varphi_{3,0}$ is unable to provide a symmetric solution, while the other Wendland functions offer much better results in terms of symmetry.

If we now inspect the results for the normal stress difference, $\tau_{p,11}-\tau_{p,22}$, we observe that when the FENE model is used (Figure 11), remarkable smoothness is retrieved by all the CSRBFs explored, but for $\varphi_{3,0}$, which somewhat underperforms at the last instants of the simulation, when the viscoelastic effects have shown up and the drop attains maximum deformation.

Figure 12 represents the normal stress difference, $\tau_{p,11}-\tau_{p,22}$, for the Hooke model. In this case, a higher degree of smoothness has a positive effect, improving the symmetry of the solution, to a certain extent. However, unless the spatial resolution of the velocity field and the velocity gradients is improved, the number of ensembles distributed over the domain is large enough, and the dumbbells per ensemble ensure a sufficiently accurate computation of the “extra-”stress at the ensembles, there is no point in further increasing the quality of the CSRBF for polymer stress reconstruction, since the extremely large, very localized stresses cannot be accurately computed unless the previous conditions are met. On the other hand, all previous conditions can be satisfied so long as the necessary computational resources are available. As a result of all the previous investigations, we find Wendland’s $\varphi_{3,1}$ CSRBF as a good choice in terms of accuracy, smoothness, and computational demands, used in this work unless otherwise noted.

#### 3.2.3. Flow Pattern under Increasing Viscoelastic Effects

**Figure 13:**
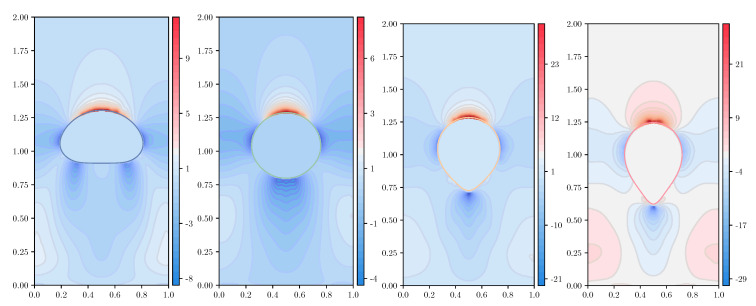
Drop shape and isocontours of normal stress difference of the polymer $\tau_{p,11}-\tau_{p,22}$ for the FENE model (b=50) with $\rho_{2}/\rho_{1}=10^{-1}=\mu_{2}/\mu_{1}$, at t=3. From left to right, c=1,De=1; c=3,De=1; c=5,De=3; and c=9,De=5.

**Figure 14:**
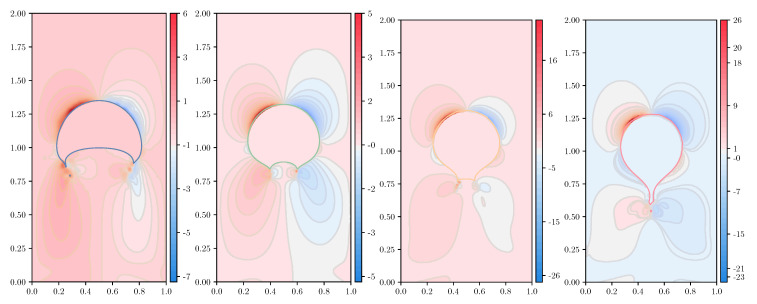
Drop shape and shear component of the polymer stress tensor $\tau_{p,12}$ for the FENE model (b=50) with $\rho_{2}/\rho_{1}=10^{-3},\mu_{2}/\mu_{1}=10^{-2}$, at t=3. From left to right, c=1,De=1; c=3,De=1; c=5,De=3; and c=9,De=5.

**Figure 15:**
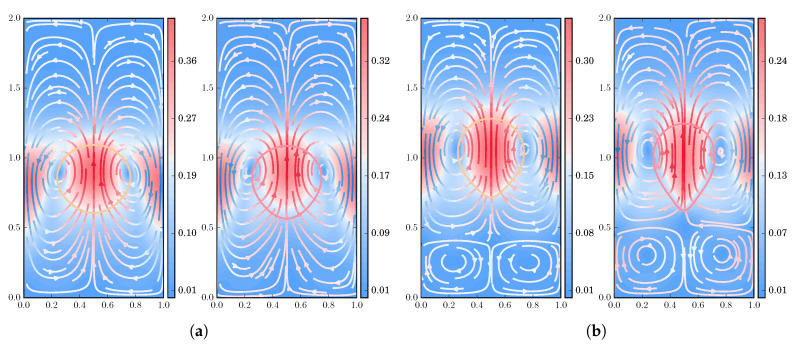
Drop shape and streamlines for the FENE model (*b* = 50), with $\rho_{2}/\rho_{1}=10^{-1}=\mu_{2}/\mu_{1}$ at different instants of time: (**a**) *t* = 2 and (**b**) *t* = 3. For each panel, the left figure (yellow line) shows the streamlines for *c* = 5, *De* = 3; the right figure (red line), those for *c* = 9, *De* = 5 (red line).

**Figure 16:**
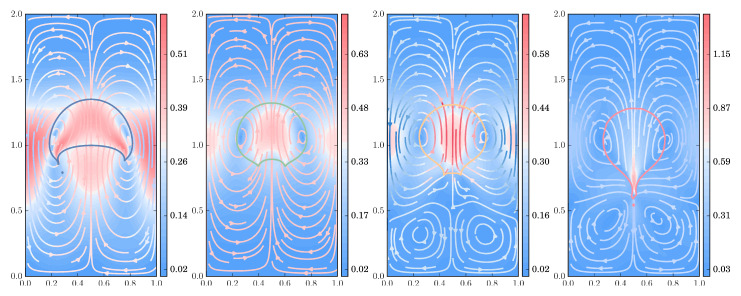
Drop shape and streamlines for the FENE model (b=50) with $\rho_{2}/\rho_{1}=10^{-3},\mu_{2}/\mu_{1}=10^{-2}$, at t=3. From left to right, c=1,De=1 (blue line); c=3,De=1 (green line); c=5,De=3 (yellow line); and c=9,De=5 (red line).

Finally, we explore the flow pattern, streamlines, and isocontours of the polymer stress tensor, in a series of simulations under increasing polymer concentration and relaxation times, using the FENE dumbbell model with extensibility parameter b=50; see Figure 13, Figure 14, Figure 15 and Figure 16. For all the subsequent numerical experiments, a uniform, unstructured mesh of size h=1/320 is used, with the number of dumbbells per ensemble $N_{d}$ and the number of ensembles $N_{ens}$ being collected in Table 1. Notice that, for stronger viscoelastic effects, a larger number of ensembles are scattered throughout the domain, reducing the computationally available number of dumbbells per ensemble, which in turn translates into a larger stochastic noise. Two different density and viscosity ratios are explored to underscore the effects caused by the polymer solution on the shape deformation and flow pattern of the drop ($\rho_{2}/\rho_{1}=10^{-1}=\mu_{2}/\mu_{1},Re=35,We=10,Fr=1$) and of the bubble ($\rho_{2}/\rho_{1}=10^{-3},\mu_{2}/\mu_{1}=10^{-2},Re=35,We=125,Fr=1$).

In Figure 13, we show the final shape and filled isocontours of the normal stress difference of the polymer stress tensor for increasing concentration of the polymer solution and longer relaxation times for the drop (density ratio of 10), while Figure 14 presents the behavior of the shear component under the same circumstances, for the bubble immersed in the polymeric media (density ratio 1000). For the drop and normal stress difference, the results present an excellent degree of symmetry and smoothness, despite the underlying stochastic procedure, even for the strongest viscoelastic effects at (c=5,De=3), which produce extreme values at the top and bottom of the rising drop.

The bubble depicted in Figure 14 with the shear stress isocontours shows how the harder computational demands of this simulation translate into a modest reduction of the overall symmetry along with more dramatic changes of the bubble shape. Nevertheless, the smoothness of the discrete solution obtained for the polymer stress tensor, even when the stronger polymer concentration is used, is such that it is possible to retrieve the maximum values at the wake of the bubble, with a high degree of accuracy and symmetry. Figure 13 and Figure 14 also evince elastic effects, with heightened drop deformation caused by larger values of the Deborah number (i.e., relaxation time of the polymer) and polymer concentration, while viscous and surface tension forces are kept at the same level.

Next, we focus on the streamlines of the flow under increasing viscoelastic effects. In Figure 15, we represent the flow pattern for the drop (moderate density and viscosity ratios), with Figure 15a presenting a snapshot at time t=2, while Figure 15b shows the pattern at the final instant of time, t=3. For these high values of polymer concentration and relaxation times, at t=3, we observe the downwards velocities that characterize the “negative wake” effect [23,76].

For large density and viscosity ratios, Figure 16 depicts the behavior of the deforming bubble at t=3. At the higher levels of polymer concentration and relaxation times, the area in which downwards velocities are observed, comprising the “negative wake”, is much larger than in the case of the deforming drop.

## 4. Conclusions

This paper presents a series of numerical experiments in an effort to gain insight into the impact that viscoelasticity may have on drop deformation, under a number of multiphase flow configurations. The numerical method employed is based on an FEM-based spatial discretization, using a semi-Lagrangian approach to deal with the convective terms, a kinetic modeling of the polymer contribution to the stress tensor, and ML-inspired techniques for building, over the whole domain, each of the components of the extra-stress tensor, which effectively decouples the “microscopic” and “macroscopic” scales. Hence, the process of mesh refinement is no longer hampered by the low number of polymer particles at a certain computational cell, allowing us to use very refined meshes and achieve excellent smoothness and accuracy in kinetic-based, complex flow simulations.

The results on drop deformation under shear flow agree extremely well with those in the literature produced by an equivalent constitutive (Oldroyd-B) model and indicate that drop viscoelasticity prevents deformation to some degree, underscoring the efficiency and viability of the stochastic approach in multiphase flows. For more demanding situations in buoyancy-driven flow, we obtain remarkable mesh (macro) and ensemble (micro) convergence for both the FENE and Hooke models; as for the degree of CSRBFs smoothness required to accurately build the “extra-”stress tensor, the numerical study points to a minimum degree of $C^{2}$ smoothness guaranteed by $\varphi_{3,1}$, with higher smoothness providing small benefits compared to the additional computational cost. Finally, we observe dramatic changes of flow pattern, including viscoelastic (“negative-wake”) effects and extremely large values of shear and normal stress differences close to the drop interface, as the polymer concentration and relaxation times increase.

In future investigations of multiphase flow of polymeric liquids, we will try to address improved resolutions and problem configurations taking advantage of this ML-enhanced method to combine isotropic and anisotropic mesh adaptation [77] with a kinetic modeling approach.

## Figures and Tables

**Table 1:** Each entry in the table shows $N_{d}\;/\;N_{ens}$ for increasing viscoelastic effects and different density ratios. $N_{d}$ is the number of dumbbells per ensemble and $N_{ens}$ the number of ensembles scattered in the domain.

Density Ratio	c=1,De=1	c=3,De=1	c=5,De=3	c=9,De=5
10	150,000/5000	75,000/10,000	50,000/15,000	37,500/20,000
1000	150,000/5000	50,000/15,000	15,000/50,000	15,000/5000

## Abbreviations

The following abbreviations are used in this manuscript:

$\mu_{1}$	Viscosity of the continuous phase
$\mu_{2}$	Droplet viscosity
$\rho_{1}$	Density of the continuous phase
$\rho_{2}$	Droplet density
$\tau_{p}$	Polymer (“extra-”)stress tensor
$\tau_{p,12}$	Shear component of the polymer stress tensor
$\tau_{p,11-22}$	Normal stress difference of the polymer stress tensor
ϕ	Level set function
φ	Compactly-supported Wendland function
χ	Support size of the CSRBF
ψ	Trial basis function
*b*	FENE extensibility parameter
*c*	Concentration parameter
c	Droplet circularity
dt	Time step size
*h*	Grid size of the uniform, unstructured mesh
*p*	Pressure field
v	Velocity field
*s*	Approximation interpolant of the CSRBF
*D*	Deformation parameter
$N_{d}$	Number of uncorrelated dumbbells per ensemble
$N_{ens}$	Number of ensembles of polymer particles
$N_{mp}$	Number of marker particles
Ca	Capillary number
Fr	Froude number
Re	Reynolds number
We	Weber number
ALE	Arbitrary Lagrangian-Eulerian method
BD	Brownian Dynamics simulations
CSRBF	Compactly-Supported Radial Basis Function
FEM	Finite Element Method
FENE	Finitely Extensible Non-linear Elastic model
LS	Level Set method
LSC	Least Squares Commutator preconditioner
ML	Machine Learning
NN	Nearest Neighbor
PBC	Periodic Boundary Conditions
PLS	Particle Level Set
RBF	Radial Basis Function
VOF	Volume-Of-Fluid method
[l c]	Vector of interpolation coefficients for the CSRBF
K	Discrete matrix system
PETSc	Portable, Extensible Toolkit for Scientific Computation
